# Contextual Complexities in Implementing a Family-Based Childhood Obesity Intervention: The Perspectives of Enrolled Children and Their Parents

**DOI:** 10.3390/children7120267

**Published:** 2020-12-02

**Authors:** Didde Hoeeg, Ulla Christensen, Louise Lundby-Christensen, Dan Grabowski

**Affiliations:** 1 Department of Health Promotion, Steno Diabetes Centre Copenhagen, 2820 Gentofte, Denmark; dan.grabowski@regionh.dk; 2 Section of Social Medicine, Department of Public Health, University of Copenhagen, 1123 Copenhagen, Denmark; ulch@sund.ku.dk; 3 Steno Diabetes Center Sjaelland, Akacievej 7, 4300 Holbaek, Denmark; lochris@regionsjaelland.dk

**Keywords:** family, children, overweight, obesity, parenting, intervention, context, qualitative

## Abstract

Family interventions to treat childhood obesity are widely used, but knowledge about how family dynamics are affected by these interventions is lacking. The present study aims to understand how a family intervention impacts the context of family dynamics, and how different contexts affect the families’ implementation of the intervention. Based on qualitative interviews, we studied families with a child between 9–12 years enrolled in a family intervention to treat childhood obesity at a pediatric outpatient clinic. We conducted 15 family interviews including 36 family members. We found that the family intervention created a new context for the enrolled children. They had to navigate in different contexts and non-supportive environments and push for change if they needed more supportive environments in their attempt to adhere to healthy habits. We show the complexities experienced by parents and grandparents when trying to comply with siblings’ and/or grandchildren’s different needs. The enrolled children were often indirectly blamed if others had to refrain from unhealthy preferences to create supportive environments. These findings are significant in understanding the important role of contexts in family-obesity interventions. This knowledge is relevant to health professionals, researchers, and policymakers.

## 1. Introduction

Childhood obesity is a major concern for public health worldwide and has been declared one of the most serious public health challenges of the 21st century [1,2]. Family-based weight management interventions have been widely used to address obesity among children and adolescents [3,4]. However, they only lead to small decreases in children’s weight [5]. This calls for more research aimed at understanding the barriers to and facilitators of weight management, so as to tailor family interventions and guide implementation [6]. Childhood obesity cannot be understood or successfully responded to without accounting for sociocultural and familial contexts. Understanding the difficult, dynamic, human complexity of familial contexts is an essential step in seeking to understand the causes, prevention, and treatment of childhood obesity [7]. However, the contexts in which family-based interventions are delivered, as well as the experiences of the families receiving treatment, are missing from the carefully controlled RCTs (randomized controlled trials) [8]. Moore and Evans (2017) argued that interventions are often viewed as discrete packages of components that are described in isolation from their context. When implementing social interventions aimed at changing a system’s dynamics, which is the case with family-based interventions, Moore and Evans called for a better understanding of the system into which an intervention is introduced [9].

Within recent intervention research, more focus has been put on understanding and including the particular context in which an intervention is supposed to work, often based on qualitative data [10,11]. Craig et al. (2018) defined context as “any feature of the circumstances in which an intervention may be implemented that may interact with the intervention to produce variation in outcomes” [10] (page 6). Thus, context can be understood as the circumstances or events that form the environment within which something exists or takes place and as that which therefore can help make the phenomena intelligible and meaningful [12]. Poland et al. (2009) distinguished between key dimensions of social context that impact three overlapping levels of health promotion work: The target phenomena, the intervention, and the evaluation of interventions [11]. The social context of the target phenomena concerns understanding the social environments in which specific behaviors are shaped or maintained in the target group. Understanding these specific behaviors can be accomplished by analyzing micro-sociological aspects of everyday life and their embeddedness in concrete contexts. The social context of the intervention entails the history of health promotion efforts in the setting and what the health promoter brings to the particular setting in relation to skills, capacity, resources, and relevant sensitivities. It is a matter of systematically “unpacking” those aspects of settings that most impact the health promotion practice [11]. Thus, understanding the relationship between interventions and the context in which they operate is critical to implementation success and failure and to whether interventions can be sustained or successfully translated from one context to another [10].

The objective of the present study was to understand how a family intervention to treat childhood obesity influenced family contexts and dynamics and to analyze how different social contexts influenced the implementation of healthier habits. We focus on the role of social contexts when a real-life family intervention (not research trial) is implemented in the families’ everyday life.

## 2. Research Setting: The Real-Life Intervention

The intervention was carried out by health professionals from the pediatric outpatient clinic at a small hospital located in a rural and disadvantaged area in Denmark. The area has a higher-than-average rate of citizens who depend on social benefits (28%) and of children living in poverty (4.6%); it has a low average life expectancy (78.5 years) as well as a low average disposable income compared with the average levels across Denmark [13]. In addition, the area has a high prevalence of overweight and obesity compared with the average rates in Denmark. Among children (14–15% years of age), 22% have overweight or obesity [14]. Among adults 55% are have overweight or obesity [15].

Children and adolescents between 3 and 17 years with BMI > +2 standard deviations above the median or with a rapid weight gain (curve crosses two major percentiles on the growth chart for children within a relatively short time period) were enrolled in the intervention. Children had been referred by their general practitioner, health nurse, or professionals at the pediatric outpatient clinic. The intervention consisted of an initial visit (1 h) followed by shorter counselling sessions (30 min) approximately every fourth to eighth week, throughout the intervention period. The families were usually enrolled for two years but sometimes for longer. At the initial visit, the child’s height, weight, and waist circumference were measured, and the family was asked to describe their wellbeing and lifestyle (covering dietary habits, physical activity, sleep routines, experiences with bullying, and wellbeing in school and at home). Afterwards, the health professional and the families discussed what kind of health behavior change should be undertaken at home before next counselling session (e.g., drinking a glass of water before eating or biking to school). At every counselling session, anthropometric measures were taken, and treatment progress was discussed. The intervention was free of charge for the families.

## 3. Methods

Interviewing the family together was considered an appropriate approach, as family interviews allow researchers to gain access to the families’ dynamics and everyday life, while still taking ethical precautions into account [16,17]. Each family interview included two to three family members. A semi-structured interview guide with different topics was used to help the family members to convey their experiences with the intervention [18]. Central topics were: (1) The counselling sessions (the initial visit and following visits): With questions focusing on how the child and parents experienced the sessions, what they talked about, who participated, and the best/worst things about the sessions; (2) the health behavior tasks: With questions focusing on what agreements were made on specific health behavior tasks, how they were decided, and when it was easy/challenging to adhere to them; (3) the families’ implementation of the intervention at home: With questions focusing on what they did with the health behavior tasks when returning home from a counselling session, did everybody in the family attempt to adhere to them, did they talk to the broader family about the intervention, how did they managed/not-manage implementing new healthy habits, how/when could family members best support, how/when could family members make it more difficult, and how/when could it be easy/challenging to adhere to healthy living in other settings (e.g., school, sport, and leisure activities, peers). All topics were made child-friendly to encourage the children to participate actively in the interview.

### Participants and Procedures

In order to obtain homogeneous data, we chose to interview families with an enrolled child between 9 and 12 years of age. This age interval was chosen because children younger than 9 years were expected to find it difficult to reflect on the interview questions and to participate in a family interview, while children older than 13 years were assumed to be more independent of their parents, rendering our focus on family dynamics difficult [19,20]. Families were excluded if they did not speak Danish fluently, if parents had severe mental disorders, or if the child lived with a foster family. The initial recruitment of families was done by the health professional, who invited families to participate and passed on information about the study and obtained approval from the families that a researcher could contact them. A researcher then arranged the interview. Twenty-nine families were contacted by the health professional, however some declined to participate, mostly due to time constraints. We used the concept of “information power” to guide an adequate sample size. Information power indicates that the more information the sample holds, relevant for the actual study, the lower the number of participants needed. This depends on (1) aim of the study, (2) sample specificity, (3) use of established theory, (4) quality of dialogue, and (5) analysis strategy. After 11 family interviews, the information power was assessed, leading us to do four additional interviews. When 15 family interviews (with a total of 36 family members) had been conducted, the researchers experienced sufficient informational power, and stopped arranging more interviews [21]. The interviews were conducted by DH and two assistants. The families had been enrolled in the intervention between 3 months and 4 years. Interviews were carried out in the families’ home, except one that was carried out at the child’s school and one at a parent’s workplace. The interviews were recorded and transcribed verbatim. The transcripts were anonymized, and sensitive characteristics were recoded to ensure anonymity. All family members received fictive names and ID numbers. Characteristics of the interviewed families are presented in Table 1.

## 4. Ethics

The study followed the codes of ethics in the Helsinki II Declaration, and it was approved by the Danish Data Protection Agency (P-2019-169). Approval from an ethics committee is not required in interview studies according to Danish legislation. Nevertheless, as the study included children, special precautions were taken to protect their rights and welfare [22]. The study was carefully designed based on ethical considerations and family interviews were deemed suitable, as children may find it uncomfortable to be interviewed alone. Further, the researchers were skilled and confident in using the family interview method [23]. To earn the trust of the families, the purpose of the study was explained in child-friendly, non-scientific language. All families gave their informed written consent. To make the health professional unrecognizable, sensitive information (e.g., name, gender, and age) as well as profession, employment unit, and hospital were anonymized.

## 5. Analysis

We used Systematic Text Condensation (STC) to analyze the data [24]. We searched the data for content concerning how families had experienced the intervention and how they had managed and implemented it in the different contexts of their everyday life. We used four analytical steps: (1) Reading the transcripts to obtain an overall impression and identify preliminary themes; (2) based on the preliminary themes, code groups were developed and meaning units reflecting the families’ implementation were identified; (3) establishing subgroups within each code group to illustrate the aspects of each code group and condensing the content of each meaning unit; (4) synthesizing the units, presenting a recontextualization, and selecting quotes to illustrate the findings. The recontextualization was based on a second-order analysis using the theory of social contexts, the goal being to reveal how the intervention had influenced the family contexts as well as how other social contexts had influenced the families’ implementation of healthier habits. The coding software Nvivo 12.0 (QSR International) was used to maintain a sense of perspective during the analysis process. A table illustrating the coding process and the second order analysis are to be found in the supplementary material, Table S1.

## 6. Results

The results will be presented in six overlapping themes illustrating: The families’ pre-intervention context (Section 6.1), the context shaped in the counselling session (Section 6.2), implementation of the intervention in family contexts (Section 6.3 and Section 6.4), the impact of other contexts on implementation (Section 6.5) and, lastly, the children’s new post-intervention context (Section 6.6).

### 6.1. Obesity as a Familial, Common and Abnormal Issue

Some of the families were aware that they lived in an area with a high level of overweight and obesity and that their local government was focused on targeting the issue.

*“(…) The municipality is really focused on the fact that there are too many overweight citizens (…) and it’s important to think about the children (…).”*

(Mother, Family F)

Several families referred to their own family as being a family that loved food and had a general weight issue among family members. *“None of us are small, we just love to eat”* (Mother, Family J). Almost all children reported having a cousin or a classmate enrolled in an intervention targeting overweight and obesity, illustrating that in this area overweight issues were common. One family reflected on how it had been difficult for the system to reach the entire family when targeting the local problem with overweight and obesity.

*“When you see a family on the street, it isn’t just a child who is obese while the others are tiny. It’s usually always an entire family with weight issues.”*

(Father, Family L)

Overall, overweight and obesity constituted a universal issue, which was also exemplified by it being a theme in mainstream TV entertainment the families watched to get inspiration about ways to be healthier. Some parents specifically tried to prevent their child from becoming severely obese by letting the child watch TV shows displaying the consequences of obesity.

*“(…) we show her (daughter) some TV shows, there are some Danish (TV) shows with extremely obese people and I can hardly watch them. It’s just an obese thing—it’s not a human being. It also scares her (daughter) a bit.”*

(Mother, Family J)

Despite the fact that overweight and obesity were perceived as a common issue among the families living in this area, this quote also demonstrates how extreme obesity was perceived as highly abnormal. To some extent, extreme obesity was somewhat dehumanized by this mother in her attempt to keep her daughter from developing severe obesity.

Several of the families had a prior history of weight intervention attempts before being enrolled in the current intervention. Previously, some of the mothers had sought help from local dietitians, but they were frustrated as they felt they had not received the support they needed to manage healthy living.

*“We took a break from the (municipal) dietitian because it didn’t go well and the kilos just increased and… we gave up (…) The only thing going on was the weighing. She (daughter) was weighed and measured and she was constantly told—and me too—don’t do that, and don’t do this, and certainly don’t do that…”*

(Mother, Family I)

For most families, the current intervention was one in a series of different intervention attempts. The intervention was characterized as being run by an impassioned health professional with limited resources assigned to counselling sessions.

### 6.2. Explaining Childhood Obesity to Children with Obesity

*“She (health professional) is so kind and open. She is always joyful and smiling. Even if you haven’t lost weight. “Then you’ll probably have next time”, “or otherwise maybe you have grown a bit”. She always has a positive approach.”*

(Mother, Family F)

Even though all families reported being very pleased with the current intervention and the parents, in general, felt the health professional was very good with children, some also experienced the health professional as being a bit too direct in communicating the child’s reason for being enrolled:

“At the first session (…) the health professional said to her (daughter) “well, you are too fat”, and I thought, is that really the way to say it?”

(Mother, Family K)

When talking about the counselling sessions, there was consensus among most children on disliking *“That you have to take your clothes off when you get on the scale”* as one child said (Child 9 years, Family C). Some children said that they participated actively in the sessions, while other children said the adults did most of the talking. What stood out for most children was that they remembered the health professional showing them narrowed veins and explaining how that could lead to blood clots and other health-related consequences of being obese.

*“(…) The more sweets you eat… and then she shows you what your veins will look like if you eat a lot of sugar and fat. (…). And if you don’t eat that much, it will look fine.”*

(Mother, Family A)

The children also recalled how they had been shown cups with crackers or candy and comparable cups showing the content of raw fat in each snack. This too had an upsetting effect on the children.

This reveals how a specific context was created at the initial counselling session with the health professional as a well-liked authority, telling the children about their obesity issue and why it was crucial to treat. However, when talking about the counselling sessions, almost all families could not recall that they had been given any specific tasks they were supposed to implement to be healthier, other than that the child should lose weight, often expressed as a certain number of kilograms.

*“We haven’t had any specific goals defined for us, other than maintaining a weight status.”*

(Mother, Family L)

Consequently, this had created a context that induced a specific focus on weight and weight loss. This was underlined by another essential pedagogical element that stood out among the children; they could pick a small toy from a giftbox if they had lost weight since the last session.

*“(…) When you have done well, then you (daughter) are allowed to pick a toy from the gift basket. At the last session she was not allowed to pick a toy because she hadn’t done well. To show you, that she (the health professional) really wants to reward you when it’s going well. But the motivation to do it even better until next time comes from knowing you will receive the reward.”*

(Mother, Family G)

One mother also reported that their daughter had received a gift for not gaining too much weight between counselling sessions when she had experienced some psychological issues during her enrolment in the intervention. Nevertheless, receiving a gift underlined that the child had acted well and deserved a gift and the opposite, not being rewarded with a gift meant that the child had not acted well (maintained or lost weight). Likewise, the children interpreted the reward as being solely based on their individual behavior between sessions. Hence, this approach created a context focused on the child’s individual responsibility in losing or maintaining weight and toned down the shared family responsibility.

### 6.3. Increased Weight Focus and Split Families

The intervention approach and activities implemented in the consultations had created a family context with an increased focus on weight and weight loss. This was evident in that the families’ implementation of the intervention in their everyday lives had become centralized on the children’s actual weight status.

*“(…) We keep an eye on her weight more often now. In fact, we have the scale under her bed”*

(Mother, Family J)

The families reported that they often measured the child’s weight at home to supplement the numbers from the pediatric outpatient clinic. Some children also mentioned monitoring their own weight without telling their parents.

*“(…) I do it secretly and I don’t tell anyone how much I weigh”*

(Child 10 years, Family H)

The weight measurement sometimes became a behavioral tool to decide whether the children could eat something unhealthy.

*“(…) I asked my mum if I could have some ice cream and then she said yes, if the weight says so. (…) And then I didn’t eat ice cream.”*

(Child 10 years, Family H)

This indicates that, in some cases, healthier choices were made based on weight status and not on general desires to be healthier. Most families reported that the child and the mother had tried to change unhealthy habits into healthier habits. Siblings were often not part of these changes, which the enrolled child often found unfair. This displayed the complex balance that had to be struck in the families’ implementation. As one mother explained, there are (physical and age-related) differences among siblings, making it challenging and not realistic for siblings to have the same dietary patterns.

*“(…) She (daughter) knows that he (her brother) is allowed to eat a bit more than she is, because he is bigger, he’s a teenager and they need more fuel and he also exercises more (…). Sometimes she thinks it’s a little unfair, but…”*

(Mother, Family F)

However, if siblings were lean, there was a clear tendency for the enrolled child and parents to perceive this as a reason why the siblings did not need to adhere to healthy eating habits. This also shows how the enrolled children linked healthiness with being slim, skinny and eating less.

*“We have chosen to do it together. Because he (son) should not be on his own in this (…). Except for his older brother. (…) His older brother is skinny.”*

(Father, Family D)

If the enrolled child had older siblings, it seemed more challenging to involve the siblings in implementing healthy habits, while it seemed less problematic when the siblings were younger than the enrolled child. Even though family members showed consideration for the enrolled child adhering to healthier habits, the family seemed to become less coherent when new family routines were created.

For example, one mother explained how siblings and the father waited to eat second and third portions, until after the enrolled child had left the dinner table.

*“David (father) eats with the others. I’m trying to (…) eat with her (daughter), right? According to her rules… but in my quantities (…) But I’m trying to follow the same principles (…) so she doesn’t feel alone in this. Her dad has a greater need for food.”*

(Mother, Family G)

Although this was done with good intentions and the enrolled girl said it was all right, it still emphasized that she deviated from the remaining family when they were split up into those who could eat several portions and those who were allowed only one portion. In general, it was challenging for the children to adhere to healthy eating when family members ate what the enrolled children were not allowed to eat.

*“When my dad drinks soft drinks and I have to stick to sparkling water, (…), it’s tough.”*

(Child 12 years, Family L)

This was also seen between siblings regarding their consummation of unhealthy snacks. Again, this was justified by referring to the siblings’ different needs based on age and body type (thinness).

*“It has been tough from time to time. Especially when his older brother can eat it without gaining weight, while Adam (son) can’t and isn’t allowed to either. (…) So, we have decided that Adam should avoid entering his older brother’s room if he can’t take it (…). We have asked his older brother to eat, what Adam isn’t allowed to eat, in his own room.”*

(Father, Family D)

The strategy the families reported often using to avoid these conflicts was for the enrolled child to stay away from the siblings’ rooms if the temptation was too much. This illustrates how complicated it was for the families to implement the intervention in their everyday lives.

### 6.4. Blamed as a Cheater or for the Healthy Spill-Over Effects on Others

This complex implementation spanned across various family contexts. In divorced families, the implementation was more complex because children resided in two households. Mothers were often frustrated with the lack of involvement of their ex-partner, experiencing that the father did not support healthy habits when the child was at his place. This made it necessary for the mothers to be even stricter in complying with healthy habits to counterbalance this pattern.

Mother: *“(…) It’s a bit difficult when you have two households. I can’t decide what they do at dad’s place. When it’s an entire week in each house, it can go really wrong (…). What he (son) loses one week, he gains in the following week, right?”* Child: *“Losing, gaining, losing, gaining…”* Mother: *“It’s a bit sad.”* Child: *“Yes…”*

(11 years, Family N)

The children were aware of this parental difference, and some children seemed to take advantage of it to eat foods that were forbidden at their mother’s place. Another perception was that the fathers behaved as they did to spoil the child when staying at his place, without giving it a lot of thought. However, in this situation, a child was still spoken about as a “cheater” for not adhering to healthy habits at her father’s place, which again emphasized the child’s responsibility.

*“(…) When he (father) eventually has Lisa, it all has to be cosy and tasty. And Lisa knows it, when they are out shopping, then they can also buy this and that… And her father cannot say “No”. She takes advantage of it, right? This and this also go into the shopping cart… So, in these situations you’re a cheater.”*

(Mother, Family H)

The health professional often tried to reach the non-supportive parent through the child and the parent attending the counselling sessions. Implicitly, this also placed on the child and the attendant parent the responsibility for involving the other parent in implementing healthier habits.

*“(…) back when Dad wasn’t so fond of vegetables, she (health professional) said that “now we have to get Dad involved” and “we need to get him to…””*

(Mother, Family N)

This meant that some children had tried to push their dads to adhere to healthier habits while at his place. Most children were also part of broader family contexts, where they often spent time. Grandparents, in particular, functioned as an extension of the family, which often complicated the children’s adherence to healthier eating.

*“Grandma has a hard time understanding that we have to be a bit careful. The other day, when she came here, she brought pastries with her - and cake.”*

(Mother, Family A)

Some grandparents managed to support their enrolled grandchild in decreasing unhealthy eating.

*“They have gotten better. Now, sweets aren’t always put on the table. Grandma also pays a lot of attention to what you are allowed to drink, so you don’t get all that sugar.”*

(Mother, Family E)

However, grandparents often created a link between several familial contexts. So, even though grandparents managed to support their grandchild in eating healthy, these changes may not automatically be perceived positively by other family members. This made it complicated for grandparents to navigate in supporting one grandchild in healthy eating and still pleasing their other grandchildren.

*“We talked to Grandma and Grandpa, (…) don’t serve so many sweets, (…) And that kinda affected all the other children and grandchildren, right? And wasn’t well received”*

(Mother, Family A)

If the other grandchildren were not happy with the grandparents’ efforts to avoid offering sweets and unhealthy snacks, the reason for the change fell back on the enrolled child.

The enrolled children were also often part of broader family contexts of cousins, aunts, and uncles. Several families reported experiencing that these extended family units took the enrolled child into account by serving healthy snacks or by asking permission to serve unhealthy snacks when spending time with the child. However, this also underlined that precautions were taken solely for the enrolled child and not aligned with preferences or general healthy behavior on the part of cousins, aunts, and uncles.

*“we pay a lot of attention to it in the entire family. Also, when she (daughter) is visiting her aunt, who has a sweet tooth (…) but they are also two thin people, who don’t think about it. (…) They always eat sweets in the evening when watching movies. (…) Then when you’re visiting, they have started to get a bowl of fruit instead or something like that”.*

(Mother, Family I)

Hence, the enrolled children were indirectly responsible when other family members had to act healthier than their preferences prescribed. Moreover, they had the responsibility for adhering to healthy eating in family contexts where more unhealthy foods were served, and nothing was changed to support the enrolled child.

### 6.5. Pushing for Change in Unsupportive Environments

All of the enrolled children spent a lot of time in various social contexts that complicated their adherence to healthy habits. Parents expressed particular frustrations when classmates, teachers, friends, and friends’ parents were unsupportive in helping their children.

*“Everyone (at lunch in class) just eats something like… there’s a friend who eats muffins, another friend got a bucket of cookies, while someone got some kind of drinkable yogurt in his packed lunch, and then I’m sitting there with my salad…”*

(Child 10 years, Family K)

Parents felt caught between letting their child eat what friends eat and making their child adhere to healthy eating while with friends. Some children were able to decline what was served to them, but found it often challenging and unfair.

*“She (a friend) knows very well that I’m not allowed to have sweets and crisps. And then this Friday to Saturday, I was at her house for a sleepover with another friend, then they and her family just had popcorn and stuff like that. And I just had to say “no, thank you.””*

(Child 10 years, Family K)

Some parents tried to push for change in other contexts and passed on healthy advice from the health professional when their child spent time there.

*“(…) When the class was on a daytrip once a week, they went to McDonalds, a pizza place or a hotdog stand (…). So, she (health professional) gave us some pamphlets, which we should pass on to the schoolteachers, (…) that it wasn’t good for him (son) (…). When they received them, they said, “We’ll read them while we are eating at McDonalds.” So, it was a bit unfortunate that we didn’t get any support there.”*

(Mother, Family N)

Some contexts fully accommodated this request, however it was still obvious that the enrolled child was the reason for these changes, as these were not the prevailing preferences and habits.

*“If you have had a sleepover at a friend’s house, we have told (the friend’s mother), that instead of serving cake in the evening, give them some fruit, when he’s visiting. And they have fully taken it in (…). So, the things we have been told by the nurse, we have just passed on.”*

(Father, Family D)

### 6.6. The Children’s New Context

When the children experienced difficulties with the intervention and being part of these social contexts, a new context emerged around them. It was obvious that the health-related consequences of ‘becoming’ obese explained in the counselling sessions had created new risk perspectives and made the children link obesity with severe diseases.

*“(…) You can get some terrible diseases …”*

(Mother, Family O)

*“For example, Cancer (…) You can die from Cancer really rapidly”*

(Child 10 years, Family O)

*“Oh, I don’t exactly know about Cancer.”*

(Mother, Family O)

This risk-perspective may have generated the motivation to undertake health behavior change. In some cases, this risk perspective was also used to show the children that they were on the right/healthy path, compared to their skinny friends who could consume unhealthy food without gaining weight.

*“She (daughter) has some friends who are really skinny girls. So, it’s really tough to watch them eat. And (the health professional) has been so good at talking with her (daughter) about it. She (health professional) pulls out the figure of blood vessels and explains what the friends’ vessels looks like, even though they don’t gain weight, and what yours (daughter) looks like.”*

(Mother, Family K)

This illustrates how the health professional attempted to help the child to deal with the experienced unfairness when spending time with friends. However, this approach still underlined the danger of unhealthy eating. The children reported that it was challenging to adhere to healthy habits when they were with friends.

*“It’s tough. Also, because as a child you get upset when all our friends are allowed… So, it can be quite a challenge.”*

(Mother, Family K)

The motivation created by offering rewards for weight loss lost its effect when the children were in these peer contexts. One boy told us how, in the beginning, his parents rewarded him with money for each lost kilo. This motivated him at first, but was entirely forgotten when he played with his friends, which was why he finally gave up on living healthy in order to be rewarded.

*“Then at one point I gave up (…) Because when I played with my friends (…) I just forgot everything about that scoreboard.”*

(Child 11 years, Family D)

Similarly, parents experienced that the children’s’ motivation had dropped when there was no weight loss, despite the repeated measurements.

*“We only put her on the scale at the sessions with the health professional. In the beginning, we did it once a week at home, but the motivation really decreased when you had just gained too much weight.”*

(Mother, Family G)

When asked, none of the children wanted to express how they felt when they were not allowed to pick a toy from the giftbox.

This illustrates how the children’s adherence to healthy habits was severely challenged, as they were mostly part of unsupportive social contexts through their entire social network, including their families, grandparents, extended families, and classmates as well as friends from sports and leisure activities.

## 7. Discussion and Conclusions

In the following, we will discuss how the current intervention interacted with family dynamics and how the intervention created a new context for the enrolled children.

### 7.1. Families as “Complex Systems”

Several parents talked about the intervention as a family matter, however our findings revealed the challenging task of implementing healthier habits in the entire family. Hawe et al. (2009) suggested that public health interventions should be viewed as critical events in the existence and history of a complex system, e.g., institutions, communities, or families [25]. Moore et al. (2019) pointed out the importance of perceiving the family as a “complex” system, explaining why social interventions within families should also be perceived as complex. When the intervention is a critical event in the history of the complex family system, it leads to the development of new structures and new shared meanings [25,26]. In the present study, a shared meaning emerged among the enrolled children and parents, which was that thinness is the healthy end point. This increased their focus on weight and created a new structure: That thinness (weight status) was perceived as an indicator of whether it was necessary to eat healthy. In conflicts between siblings, ‘thinness’ (together with age) was used as an argument for why older siblings could eat as unhealthily as they wished, as long as they did not do it in front of the enrolled child. The intervention under study did not engage key family members in supporting the enrolled child and uniting the family. These findings are in accordance with previous research showing that it is difficult to engage family members in family interventions [23,27,28] and that parents find it difficult to implement healthy habits if siblings do not need to lose weight [29,30]. A vast amount of research has been done on the relation between family factors, childhood obesity, and success with weight loss through validated quantitative measures [31,32,33]. Poor family functioning is associated with an increased risk of childhood obesity. However, a greater understanding of the mechanisms behind this relationship is needed [32]. Little is known about how information gathered by the participating families is translated at home and assessments of family dynamics and the relation to child weight management may provide new insights into the difficulties of pediatric weight management, lack of weight status improvement, and may provide new avenues to address issues of weight in children [31,32,33]. The present study with its sociological qualitative approach adds to the existing research by revealing the social mechanisms that are triggered when a child is enrolled in a family intervention that interacts with the pre-existing dynamics prevalent in each family.

### 7.2. The Impact of Unsupportive Social Contexts

The analysis showed that when the intervention interacted with the different social contexts in which the children spent their everyday lives, this occurred through the child and family. The enrolled children found it difficult to adhere to healthy habits when they visited members of the extended family and grandparents, or were in school, sports, and leisure time settings as well as when visiting friends and their families. General preferences for unhealthy eating seemed to manifest in these different contexts and be part of the “social context of the phenomena”. The health professional attempted, in some cases, to help the child and parent by suggesting strategies for encouraging others (fathers, grandparents, teachers, or classmates) to move in healthier directions and create supportive healthy environments. This illustrates how the “social context of the intervention” attempted to affect the “social context of the phenomena” through the enrolled child and attending parent. Sometimes this went well and at other times it led to counter-pressure that the enrolled child or parent had to deal with. If changes were made in healthier directions to support the enrolled child, it was still obvious that this child was the reason for the changes. In this way, the enrolled child was indirectly “guilty” for the healthy changes others had to make by refraining from their preferences. This emphasized the perception that children with obesity were expected to live healthily, while non-obese children and adults did not require the same healthy eating habits. Moore et al. (2019) argued that children and parents are active agents, whose behavior continuously adapts in response to feedback from one another, generating patterns of behavior in the family [26]. Our study showed how these family dynamics were continuously affected by other contexts with which the family and child interacted. This finding is in accordance with the argument that parents’ actions cannot easily be isolated from broader family and community systems [26]. If the behaviors of families are perceived as interrelated with the context in which they live, it may be difficult for an individual family member to change unhealthy practices [34]. In the current case, the enrolled children and attending parent became responsible for creating healthy environments around themselves, which was a difficult task, primarily because it was the overweight per se that was the main problem—not unhealthy living as such. We argue that it is vital to pay greater attention to the socioecological context of childhood obesity. This is in line with earlier research [7,8]. Fiese et al. (2013) argued that whether or not a given intervention will have an effect is influenced by variations in family structure and routines, cultural traditions, and community resources as well as the child’s self-regulation—highlighting the notion that context matters in childhood obesity [8].

### 7.3. The Children’s New Context

The present study showed how being enrolled in the intervention created a new context for the children and gave them a complex role to navigate in: (1) They were labelled as obese and experienced an increased focus on their weight status. Several children disliked taking their clothes off when going on the scale or when their waist circumference was measured, which could indicate a feeling of shame about their body. (2) They had begun to equate healthiness with thinness when presented with the health-related consequences of obesity. They tried to act based on this new information, even though it was often in conflict with their family members’ behavior. (3) The children (and their parents) remembered receiving limited guidance on actual behavior change, while the children were indirectly given responsibility for weight loss through a system of rewards. (4) The families were often divided into those who needed to adhere to new healthy habits and those who did not. The children often experienced less support from some family members (often fathers and siblings), while they risked being referred to as “cheaters” if they did not adhere to healthy habits when spending time in unsupportive family contexts. The parents often had to follow a complicated parenting strategy to meet the different needs of siblings. The enrolled children endured this, despite often finding it unfair. Earlier studies have shown that conflicts and frictions often emerge when families implement a family-based intervention [23,27,35], however in the present study, conflicts did not seem to occur to the same degree. The children seemed to accept that they had to adhere to new healthier habits even when other family members did not. This may be because the children perceived themselves as responsible for losing weight and did not necessarily consider the intervention to be family based. (5) In some cases, the enrolled children and attending parent(s) had assumed responsibility for pushing others to be healthier, creating in the way supportive environments around themselves. Furthermore, the children risked being indirectly blamed if family members had to refrain from their preferences for unhealthy eating. Eg et al. (2017) also reported on how siblings expressed irritation that their sister’s or brother’s efforts to reduce intake of unhealthy food had negative spill-over effects on them [27]. The situation also forced grandparents to choose between treating all their grandchildren equally and pleasing some of them. In supportive family environments, it was still obvious that changes had been made because of the enrolled children, which again rendered them the cause of the healthy changes. (6) The children found it difficult and unfair to have to adhere to healthy eating when they were with classmates, friends, and friends’ families, demonstrating how parents had to strike a balance between letting their child do what their friends did and trying to encourage these contexts to be healthier. Despite the fact that the children were part of several unsupportive environments, they were still expected to adhere to healthy habits. This was stressed through the system of rewards, which emphasized the child’s responsibility for achieving weight loss. This may also have reinforced the children’s belief that their obesity was their own fault.

All of the above illustrates that the new context had become evident for the enrolled children and given them a new complex role to navigate. Such a new role may cause children to see themselves as deviant and the obesity as self-induced. Furthermore, if enrolled children feel they are the reason others have to abstain from their preferences, this may cause them to feel their presence is inconvenient. Further, when children perceive that they are responsible for living in a healthy manner, while not achieving the expected weight loss, they may negatively influence their psychological well-being. Children who fail to lose weight and believe they are responsible for their weight status are vulnerable to decreased self-esteem [36]. This illustrates that ethical considerations are extremely important in relation to family interventions designed to treat childhood obesity. When running public health interventions, it is important to be aware of the risk of unintended effects. Well-intended interventions may also have unintended adverse effects. Obesity interventions targeting the individual are known to carry the risk of stigmatizing participants [37], while socio-psychological harms are rarely considered in evaluations of interventions [38]. Based on our findings, it is also important to consider the children’s increased attention to their weight status, as the onset of eating disorders often occurs in childhood or adolescence, with body dissatisfaction and unhealthy weight loss behaviors as shared risk factors [39]. A newly published study found that interventions focused on weight itself were experienced as demotivating, while the health professionals’ awareness of the contextual complexity of obesity was found to be important when guiding families [40]. To our knowledge, this study is the first to use the sociological theory of social contexts developed by Poland et al. (2009), to unfold how a family intervention to treat childhood obesity influenced family contexts and how different social contexts influenced the implementation of healthier habits [11]. This study of the experiences of children and their families contribute to the existing literature by shedding light on three vital and concerning aspects of family interventions to reduce childhood obesity. Firstly, it highlights the importance of how research trials of family interventions to reduce childhood obesity may become translated into real-life interventions in everyday healthcare settings. Secondly, it emphasizes how contextual complexities can make it extremely challenging for families’ to implement a family intervention in their everyday lives. Thirdly, it illustrates how well-intended interventions risk to induce unintended outcomes that might do more harm than good among the enrolled children.

## 8. Strengths and Limitations

The family interviews contributed in-depth data, which were valuable when examining how the family intervention was implemented and managed in real-life settings and how different contexts impacted the children’s adherence to healthy habits in order to lose weight. We experienced that using family interviews as a method helped us to create a comfortable interview setting for both the children and their parents. However, the method posed the risk that children or parents would not express themselves fully due to the presence of other family members as they might not feel free to honestly state their opinions [19]. The researchers were aware of this risk and tried to encourage all family members to convey their experiences and perspectives, while being sensitive to not pushing children and parents when something seemed unpleasant to talk about (e.g., none of the children wanted to talk about how they felt when they did not receive a gift).

Because the health professional from the intervention helped to recruit the families for an interview, there is a potential risk of selection bias. Although the families expressed being very pleased with the health professional, they had both positive and negative things to say about the intervention.

## 9. Implications for Practice and Future Research

Our study helps to fill the gap in the research literature on adverse effects of behavioral family interventions targeting childhood obesity [5] by letting the qualitative data display the social mechanisms caused by an intervention. We suggest that more research should focus on investigating the risks of adverse effects when childhood obesity is targeted through behavioral family interventions. Further, we propose that interventions should guide the child and family in a way that acknowledges that health behavior change in families is complex. We underline the importance of carrying out thorough ethical reflections on the unintended effects that may be caused by interventions designed to treat childhood obesity. When interventions to treat obesity are conducted through pediatric outpatient clinics, this may reinforce the perception that the child is the subject of the intervention and downplay the family perspective. Furthermore, health professionals from pediatric outpatient clinics may lack the resources and tools to involve the whole family and to impact the family dynamics. We suggest that policymakers be aware that it may not be enough to involve families in treating childhood obesity. Instead, perhaps the target should be the broader contexts in which families’ and children’s lives unfold.

## Figures and Tables

**Table 1:** Family characteristics.

	Number	Total
**Children**		15
Female	10	
Male	5	
**Age**		
9 years	6	
10 years	4	
11 years	4	
12 years	1	
**Parents**		21
Mother	14	
Father	7	
**Family members**		36

## Supplementary Materials

The following are available online at https://www.mdpi.com/2227-9067/7/12/267/s1, Table S1: Overview of the coding process and the second order analysis.
