# Supplementary material for: Contextual Complexities in Implementing a Family-Based Childhood Obesity Intervention: The Perspectives of Enrolled Children and Their Parents
Source: Children (Basel). 2020 Dec 2;7(12):267. doi: 10.3390/children7120267 (PMC7761258; doi:10.3390/children7120267)
Supplement: Supplementary file 1 [file children-07-00267-s001.pdf]

Table S1. Overview of the coding process and the second order analysis.

Code groups	Subgroups	Second order analysis, theory of social contexts	Recontextualisation
**The counselling sessions at the paediatric outpatient clinic**	- The parent's immediate experiences<br>- The children's immediate experiences<br>- Being measured and going on the scales<br>- Telling the children about their obesity<br>- The consequences of being obese (risk focus)<br>- Tools: raw amount of fat in various snacks.<br>- The rewarding<br>- Health behaviour tasks<br>- The time interval between sessions<br>Family participation in the counselling session	**Theme 1:** The families´ pre-intervention context (section 6.1).<br>**Heading 1**: *"Obesity as a familial, common and abnormal issue"*	**The family's everyday lives**<br>- Parent perceptions of the obesity issue in their living area<br>- Family obesity narrative<br>- Knowing other children/families with obesity issues<br>- Experiences with earlier obesity-interventions<br>The obesity issue in the media
**The family's everyday lives**	- Parent perceptions of the obesity issue in their living area<br>- Family obesity narrative<br>- Knowing other children/families with obesity issues<br>- Experiences with earlier obesity-interventions<br>The obesity issue in the media	**Theme 2:** The context shaped in the counselling session (section 6.2),<br>**Heading 1:** "Explaining childhood obesity to children with obesity"	**The counselling sessions at the paediatric outpatient clinic**<br>- The parent's immediate experiences<br>- The children's immediate experiences<br>- Being measured and going on the scales<br>- Telling the children about their obesity<br>- The consequences of being obese (risk focus)<br>- Tools: raw amount of fat in various snacks.<br>- The rewarding<br>- Health behaviour tasks<br>- The time interval between sessions<br>Family participation in the counselling session
**The family's management and implementation of healthier habits**	- Missing health behaviour tasks<br>- Family habits and habits of the parents<br>- The role of siblings<br>- Divorced families<br>- The role of dad<br>- Implementation strategies and new rules<br>- Rewarding<br>- Weight focus<br>Conflicts	**Theme 3:** Implementation of the intervention in family contexts (section 6.3, 6.4)<br>**Heading 3:** "Increased weight focus and split families"<br>**Heading 4:** "Blamed as a cheater or for the healthy spill-over effects on others"	**The family's management and implementation of healthier habits**<br>- Missing health behaviour tasks<br>- Family habits and habits of the parents<br>- The role of siblings<br>- Divorced families<br>- The role of dad<br>- Implementation strategies and new rules<br>- Weight focus<br>- Conflicts<br>**The role of the broader family**<br>- The role of grandparents<br>- The role of aunts, uncles and cousins,<br>Support and preferences
**The role of the broader family**	- The role of grandparents<br>- The role of aunts, uncles and cousins<br>- Support and preferences	**Theme 4:** The impact of other contexts on implementation (section 6.5)<br><br>**Heading 5**: "Pushing for change in unsupportive environments"	**The role of other settings**<br>- At school<br>- At sport and leisure activities<br>- Spending time with friends and friends' families
**The role of other settings**	- At school<br>- At sport and leisure activities<br>- Spending time with friends and friends' families	**Theme 5:** The children´s new post-intervention context (section 6.6)<br><br>**Heading 6:** "The children's new context"	**The counselling sessions at the paediatric outpatient clinic**<br>- The consequences of being obese (risk focus)<br>- The rewarding<br>**The role of other settings**<br>- At school<br>- At sport and leisure activities

	- Spending time with friends and friends' families<br>**The family's management and implementation of healthier habits**<br>- Implementation strategies and new rules<br>- Rewarding<br>Weight focus
